# A Case of a 31-Year-Old Female Patient With Systemic Lupus Erythematosus Presenting With Lupus-Associated Pleural Effusions and Newly Diagnosed Lupus Nephritis

**DOI:** 10.7759/cureus.104625

**Published:** 2026-03-03

**Authors:** Khadyoth S Nanneboyina, Juan Avila

**Affiliations:** 1 Internal Medicine, Methodist Dallas Medical Center, Dallas, USA

**Keywords:** autoimmune, lupus nephritis, pleural effusion, pulmonary sle manifestations, systemic lupus erythematosis

## Abstract

Systemic lupus erythematosus (SLE) is a chronic autoimmune disease characterized by multiorgan involvement and the presence of antinuclear antibodies (ANA). Given the potential for multiorgan involvement, patients with SLE can present with a variety of phenotypes. The most common presenting symptoms of SLE are constitutional, such as fevers, fatigue, malaise, and weight loss. This is followed in prevalence by cutaneous and musculoskeletal manifestations. Pulmonary, renal, and gastrointestinal manifestations occur at similar rates of prevalence. Workup of SLE involves checking autoantibodies and complement levels. Management of SLE is highly complex and varies based on disease severity as well as clinical presentation. Current guidelines recommend the use of hydroxychloroquine in all patients with SLE (unless contraindicated) along with other immunomodulators. In this article, we present the case of a 31-year-old woman with previously diagnosed SLE who presented with both pulmonary and renal manifestations. We discuss the disease progression, as well as an overview of the management for these manifestations.

## Introduction

Systemic lupus erythematosus (SLE) is a chronic autoimmune disease characterized by multiorgan involvement and the presence of antinuclear antibodies (ANA) [1, 2, 3]. Given the potential for multiorgan involvement, patients with SLE can present with a variety of phenotypes, including but not limited to constitutional, cutaneous, musculoskeletal, hematologic, vascular, pulmonary, gastrointestinal, and neuropsychiatric manifestations [1, 2]. The most common presenting symptoms of SLE are constitutional, such as fevers, fatigue, malaise, and weight loss [1, 2]. This is followed in prevalence by cutaneous and musculoskeletal manifestations such as discoid lupus rash, verrucous rash, malar rash, photosensitivity, urticaria, polyarthritis, avascular necrosis (usually of the hip joints), and myositis [1, 2]. Pulmonary manifestations of SLE include exudative pleural effusions, pleuritis, interstitial lung disease, acute lupus pneumonitis, diffuse alveolar hemorrhage, and pulmonary hypertension [1, 2, 4]. Renal manifestations include lupus nephritis, proteinuria, and hematuria [1, 2]. Gastrointestinal manifestations of SLE include peritonitis, enteritis, and hepatosplenomegaly [1, 2]. Neuropsychiatric manifestations include headaches, stroke, and psychosis [1, 2].

The diagnostic approach to SLE involves complete blood count (CBC), comprehensive metabolic panel (CMP), inflammatory markers (C-reactive protein, erythrocyte sedimentation rate), and urinalysis [1, 2, 3]. In patients with suspected SLE, autoantibodies and complements such as ANA, anti-double-stranded DNA antibody (anti-dsDNA Ab), antiphospholipid antibody (APL Ab), lupus anticoagulant, anticardiolipin antibody, complement 3 (C3), and complement 4 (C4) should be obtained [1, 2, 3]. ANA is positive in ~97% of SLE cases and has high sensitivity (95-97%); however, it should be noted that ANA has low specificity for SLE (20%) [1]. Anti-dsDNA Ab also has high sensitivity for SLE (~96%) and low-moderate specificity (52%-70%) [1]. C3 and C4 levels are often decreased in SLE [1, 2, 3]. When combined with positive ANA, the presence of low C3 and C4 has high specificity for SLE (~94%) [1, 2]. The 2019 European League Against Rheumatism/American College of Rheumatology (EULAR/ACR) classification criteria serve as a major framework for the workup of SLE. This guideline also accounts for the presence of anti-beta-2 glycoprotein antibody (to assess for concurrent antiphospholipid syndrome) and anti-Smith antibody [3]. In some cases, anti-Ro (SSA) and anti-La (SSB) antibodies may also be present in patients with SLE [1, 2].

Management of SLE is highly complex and varies based on disease severity as well as presentation. Current guidelines recommend the use of hydroxychloroquine in all patients with SLE (unless contraindicated) along with other immunomodulators, such as glucocorticoids, mycophenolate mofetil (MMF), azathioprine, and methotrexate [1, 2, 3, 5]. Advanced therapies also include rituximab (anti-CD20 monoclonal Ab), belimumab (anti-B lymphocyte stimulator protein receptor Ab), and anifrolumab (anti-Type 1 interferon receptor Ab) [1, 5, 6]. In this article, we present the case of a 31-year-old woman with previously diagnosed SLE who presented with both pulmonary and renal manifestations. We discuss the disease progression, as well as an overview of the management for these manifestations.

## Case presentation

**Figure 1:**
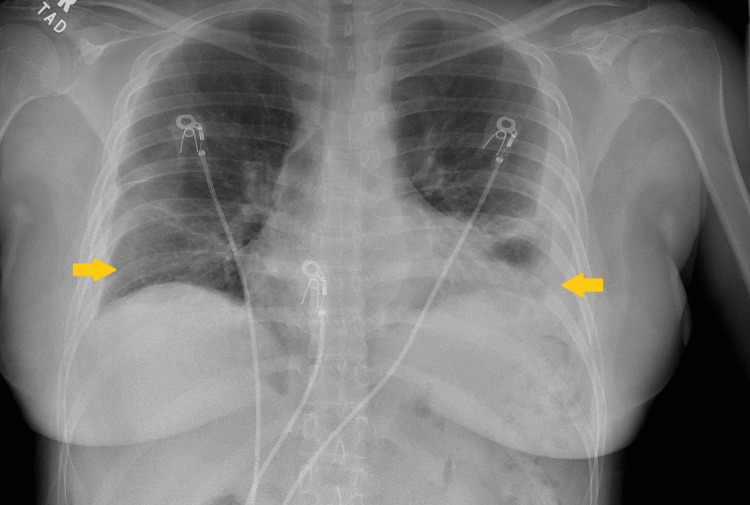
A chest X-ray from the initial hospitalization showed a small right-sided pleural effusion and a moderate left-sided pleural effusion.

**Figure 2:**
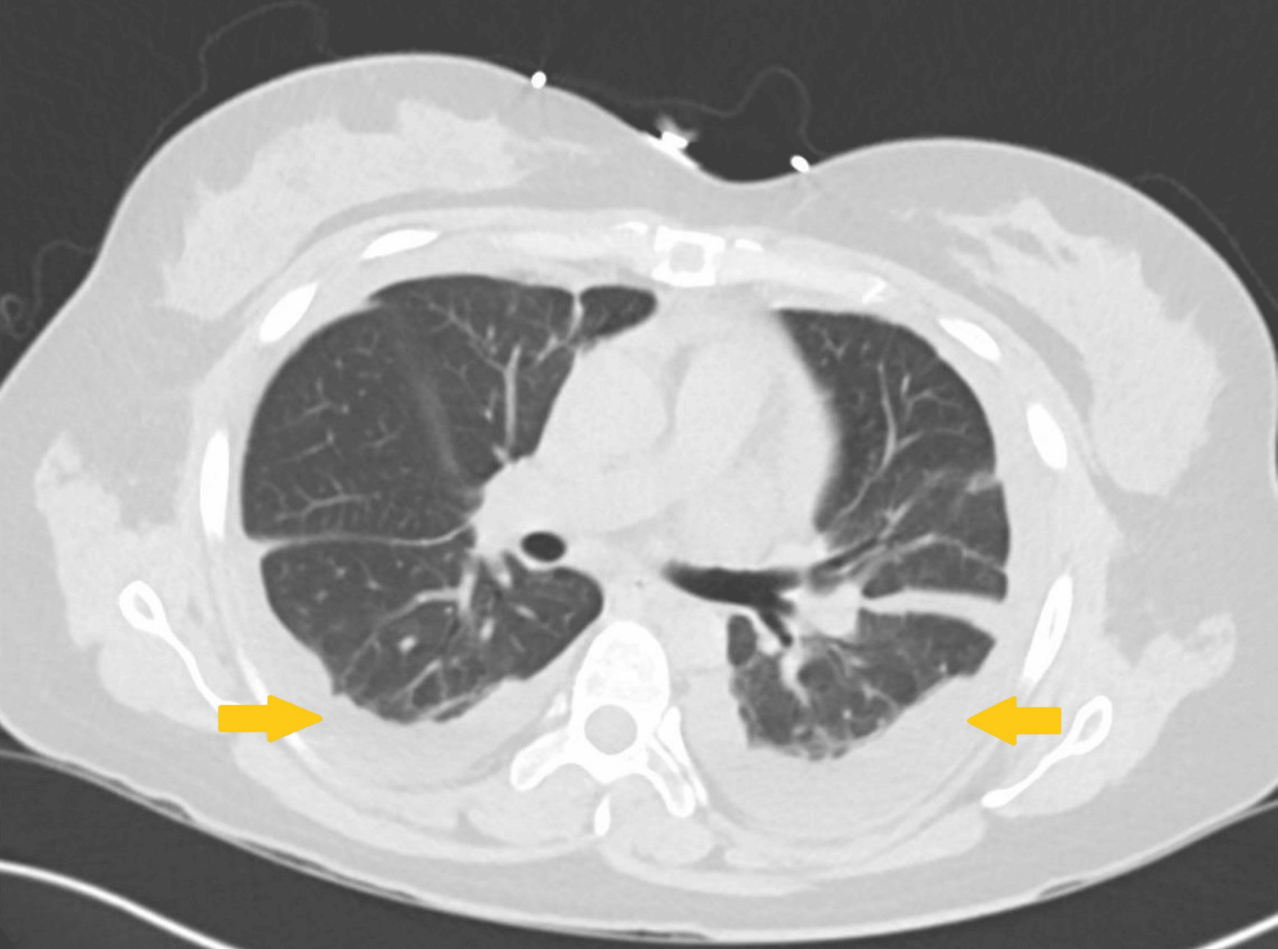
A CT scan of the chest without contrast from initial hospitalization showed bilateral pleural effusions.

A 31-year-old woman with a past medical history of SLE (on prednisone and MMF) and prior pericardial effusion status post pericardial window presented to the hospital for left upper shoulder pain. The patient reported the pain as 9/10 in intensity and worse with inspiration. She stated that it was not associated with dyspnea. Vital signs on presentation were temperature 98.3°F, heart rate (HR) 98 beats/min, blood pressure (BP) 143/83 mmHg, respiratory rate (RR) 20 breaths/min, and oxygen saturation (SpO₂) 100% on room air (Table 1). The initial physical exam was unremarkable, including a cardiopulmonary exam. Initial labs showed WBC 3.7 (x10^3)/uL, Hgb 11.8 g/dL, and platelets 471 (x10^3^)/uL; the differential diagnosis was unremarkable, including a lack of neutropenia. Additional admission labs showed total protein of 5.9 g/dL and albumin of 2.8 g/dL. C3 and C4 levels were noted to be 47 mg/dL and <8 mg/dL, respectively (Table 2). Chest X-ray on admission showed bilateral pleural effusions (left greater than right) (Figure 1). This was followed by computerized tomography (CT) of the chest without contrast, which showed small bilateral pleural effusions (Figure 2). Given the patient’s imaging findings and negative infectious workup, a rheumatology consult was placed due to concern for a possible SLE flare. The patient’s bilateral pleural effusions were thought to be secondary to SLE-associated serositis in the setting of an SLE flare, which was supported by the low C3 and C4 levels. The patient was discharged on prednisone and MMF and referred to follow-up outpatient with rheumatology at discharge.

**Table 1:** The patient's vital signs at admission

Vital Sign	Value	Reference Range	Units
Temperature	98.3	97-100.4	Fahrenheit
Heart rate	98	60-100	beats/minute
Blood pressure	143/83	<120/<80	mmHg
Respiratory rate	20	12 to 20	breaths/minute
SpO_2_	100	>90	%

**Table 2:** Selected admission laboratory results

Laboratory	Value	Reference Range	Units
White blood cell (WBC) count	3.7	3.8-11	(x10^3)/μL
Hemoglobin (Hgb)	11.8	12.0-16.0	g/dL
Platelets	471	130-400	(x10^3)/μL
Total protein	5.9	6.0-8.0	g/dL
Albumin	2.8	3.5-5.7	g/dL
C3	47	88-165	mg/dL
C4	<8	14-44	mg/dL

**Figure 3:**
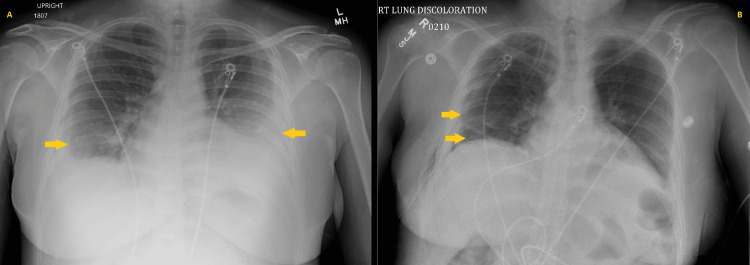
Chest radiographs obtained on readmission one month after the initial hospitalization showed worsening bilateral pleural effusions compared to day 9 of rehospitalization (A) and following right-sided decortication with placement of two chest tubes (B).

**Figure 4:**
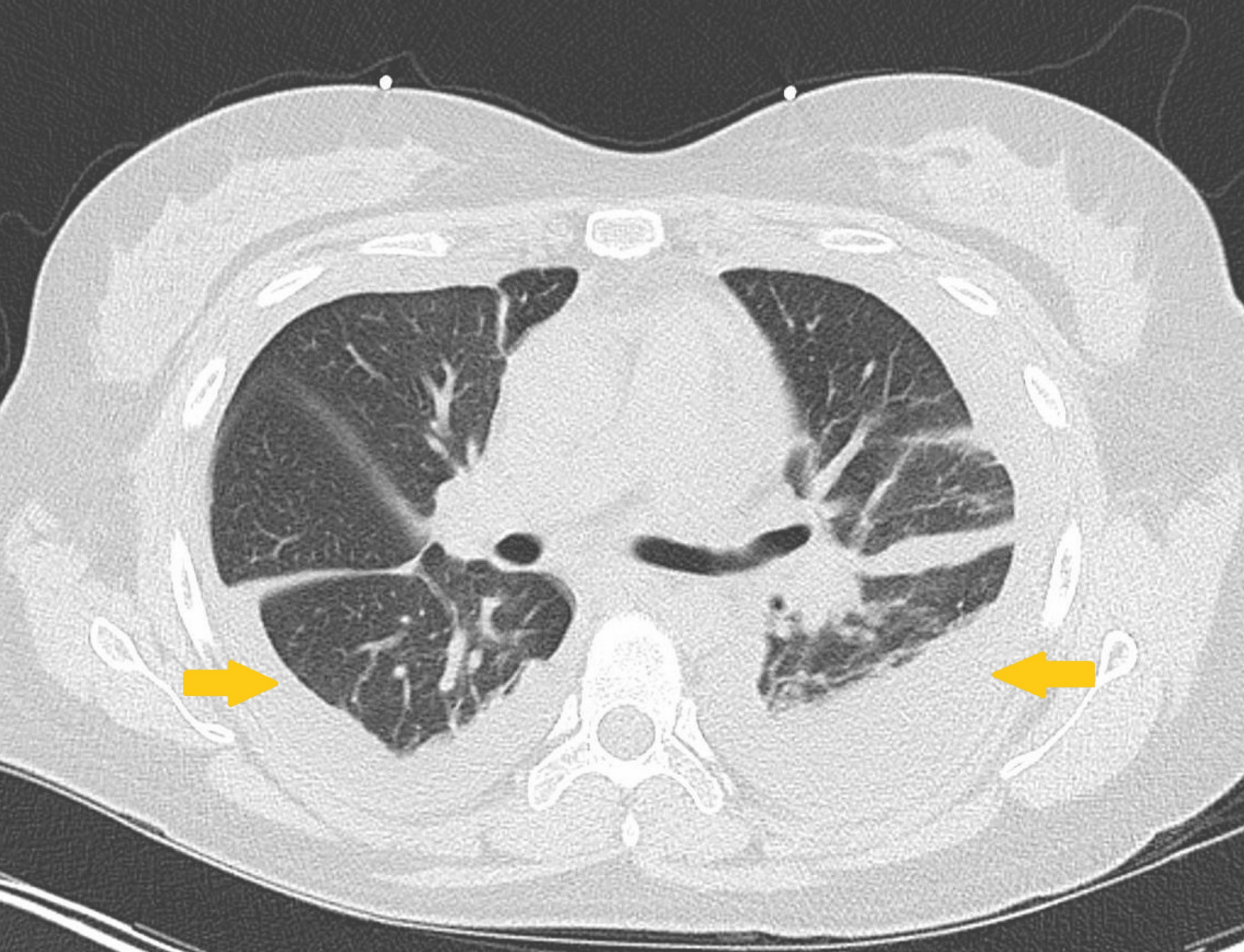
A CT of the chest without contrast was done on re-admission one month after initial hospitalization, showed worsening bilateral loculated pleural effusions.

Approximately one month after discharge, the patient returned to the ED due to a one-day history of retrosternal chest pain that was intermittent in nature and 8/10 in intensity; denied associated dyspnea or palpitations. Vitals at this time were temperature 99.1°F, HR 96 beats/min, BP 121/78 mmHg, RR 18 breaths/min, and SpO₂ 95% on room air. The admission physical exam was notable for decreased breath sounds over the left lower lung base. Admission labs showed WBC 5.5 (x10^3)/uL, Hgb 11 g/dL, platelets 577 (x10^3)/uL, total protein 6.0 g/dL, and albumin 2.7 g/dL; all other admission labs were unremarkable. Chest X-ray on admission showed worsening bilateral pleural effusions (left still greater than right) (Figure 3). A CT of the chest without contrast showed bilateral loculated pleural effusions (Figure 4). On day 2 of hospitalization, the patient was noted to be hypoxic with SpO₂ 88% on room air; diuresis was initiated along with supplemental oxygen. An interventional radiology consult was placed, given the underlying bilateral loculated pleural effusions. Pleural fluid analysis showed no WBCs and no organisms seen on Gram stain. On day 3 of hospitalization, the patient was noted to have a maximum temperature (Tmax) of 102°F and HR of ~130 beats/min, concerning for sepsis. Empiric piperacillin-tazobactam and azithromycin were started for coverage of possible pneumonia. An infectious disease consult was placed due to persistent fevers and lack of organisms seen on pleural fluid analysis, which resulted in narrowing of antibiotic coverage to ceftriaxone and azithromycin. Despite completion of five days of antibiotic coverage for community-acquired pneumonia, the patient continued to have a fever to a Tmax of 102.2°F over the next several days of hospitalization; antibiotics were extended. A rheumatology consult was placed, who recommended checking anti-ds DNA Ab and initiation of hydroxychloroquine. Anti-dsDNA Ab was noted to be markedly positive once again at >1:2560, indicative of an ongoing SLE flare. The patient was started on pulse-dose steroids due to suspected cyclical fevers secondary to SLE flare; antibiotics were discontinued.

The thoracic surgery service was also consulted during the hospitalization due to persistent right-sided pleural effusions, and the patient was taken for right-sided total thoracoscopic decortication. Following thoracoscopic decortication, the patient was transferred back to the floor with the insertion of two chest tubes, which were eventually removed (Figure 3). Additional rheumatologic evaluation was undertaken, which noted the presence of hematuria and proteinuria on several protein urinalyses, which was concerning for possible renal involvement of SLE. The nephrology service was consulted due to concern for lupus nephritis and stage 1 acute kidney injury (creatinine 0.9 mg/dL; baseline 0.5-0.6 mg/dL); a recommendation was made for renal biopsy. Pathology review of the renal biopsy confirmed combined class IV (diffuse) and class V (membranous) lupus nephritis with 30% cellular crescents. The patient’s dose of MMF was increased, and a prednisone taper was initiated with a plan for six months of induction therapy. Full-dose anticoagulation with enoxaparin was also started due to nephrotic-range proteinuria. The patient was discharged with scheduled outpatient rheumatology, nephrology, and pulmonology follow-ups.

## Discussion

As mentioned previously, pulmonary manifestations of SLE include lupus pleuritis, pleural effusions, acute lupus pneumonitis, interstitial lung disease, and diffuse alveolar hemorrhage [1, 2, 4]. Rarer pulmonary manifestations include pulmonary emboli, pulmonary hypertension, and shrinking lung syndrome [4]. In the case above, the patient presented with worsening bilateral pleural effusions concerning for SLE-associated pleural effusions. Per the 2019 EULAR/ACR guidelines, diagnosis of SLE-associated pleural effusions only requires radiologic evidence of pleural effusions (given that alternative causes of pleural effusion have been ruled out) [3, 4]. The management of choice for these high-dose steroids and response is usually brisk, with rapid improvement in pleural effusions [4, 5, 6]. Effusions that are refractory to steroids or loculated can be managed with pleurectomy or decortication, as was the case with this patient [4].

The patient’s clinical course was also complicated by a biopsy-proven diagnosis of lupus nephritis. Lupus nephritis is a major complication of SLE, with nearly half of patients with SLE developing it, and it is the most common cause of kidney injury in patients with SLE [7]. Patients with SLE who develop lupus nephritis on average develop it earlier in the disease course (first six to 36 months) [7]. More aggressive lupus nephritis and progression to kidney failure are associated with male sex, African-American and Hispanic heritage, and the presence of multiple autoantibodies [7]. Of those who develop lupus nephritis, approximately 10%-30% progress to kidney failure requiring renal replacement therapy [7]. The gold standard for the diagnosis of lupus nephritis is a kidney biopsy [7]. Clinical suspicion for lupus nephritis should be increased in patients with SLE who present with proteinuria >500 mg/day, decreased renal function, and the presence of casts (especially RBC or WBC casts) [7]. Based on kidney biopsy pathology, lupus nephritis is then classified as Class I (minimal mesangial lupus nephritis) through Class VI (advanced sclerosis) [1, 2, 5, 7]. These classes are further subdivided into non-proliferative lupus nephritis (Class II, V) and proliferative lupus nephritis (III, IV, V) [7]. While the various nuanced induction regimens for treatment of the different classes of lupus nephritis are out of the scope of this article, the overview of induction therapy involves high-dose steroids (IV methylprednisolone 0.25-1 g/day (x1-3 days)) followed by a prolonged prednisone taper [7, 8]. Other medications commonly used for induction include MMF and cyclophosphamide [7, 8].

In the patient above, the induction regimen involved both methylprednisolone and MMF. Following the transition to oral prednisone for a prolonged taper, the patient was also initiated on trimethoprim-sulfamethoxazole (TMP-SMX) due to being on >20 mg of daily prednisone for >1 month [7]. Initiation of TMP-SMX is especially important in patients with lupus nephritis undergoing induction therapy for pneumocystis prophylaxis. The patient was also started on therapeutic anticoagulation with enoxaparin due to low albumin levels. Although the patient presented with albumin levels of 2.7 g/dL, her albumin was noted to reach a nadir of 1.8 g/dL and stabilize between 2.3 and 2.5 g/dL over the rest of the hospital course. Recent guidelines and studies suggest that anticoagulation with enoxaparin or warfarin is indicated for nephrotic syndrome and albumin <2 g/dL (given that bleeding risk is intermediate) [8, 9]. Anticoagulation with enoxaparin or warfarin is also indicated for nephrotic syndrome and albumin of 2-2.5 g/dL (given that bleeding risk is low) [8, 9]. Data on the use of direct oral anticoagulants, such as apixaban, are limited and still evolving [9].

## Conclusions

In this article, we present the case of a 31-year-old female with a prior diagnosis of SLE with new-onset SLE-associated pleural effusions and biopsy-proven lupus nephritis. We illustrated the various phenotypic presentations of SLE with a special emphasis on pulmonary and renal manifestations, along with management strategies. The backbone of management involves high-dose steroids and other immunomodulators such as MMF, azathioprine, methotrexate, and cyclophosphamide.
